# Diffuse Large B-cell Lymphoma With Tumor Thrombus in the Superior Mesenteric Vein: A Case Report

**DOI:** 10.7759/cureus.117570

**Published:** 2026-10-06

**Authors:** Taisei Katahira, Yuji Ohizumi, Masaaki Akahane, Naoki Yoshioka, Yuichiro Hayashi, Shigeru Kiryu

**Affiliations:** 1 Department of Radiology, International University of Health and Welfare Narita Hospital, Chiba, JPN; 2 Department of Pathology, International University of Health and Welfare Narita Hospital, Chiba, JPN

**Keywords:** 18f-fdg pet/ct, computed tomography, difuse large b cell lymphoma, superior mesenteric vein, tumor thrombus

## Abstract

Intravascular tumor thrombus, also referred to as tumor thrombus, is a well-recognized finding in various solid organ malignancies. Although rare, there have also been several case reports of tumor thrombus in malignant lymphoma. We report a case of diffuse large B-cell lymphoma (DLBCL) presenting as a mesenteric mass with tumor thrombus involving the superior mesenteric vein (SMV). A 76-year-old woman presented with abdominal pain. Contrast-enhanced computed tomography (CT) revealed a well-defined, lobulated mesenteric mass with heterogeneous enhancement. A tumor thrombus extending into the SMV was also identified. Fluorine-18 fluorodeoxyglucose positron emission tomography/CT demonstrated intense fluorodeoxyglucose uptake in both the mesenteric tumor and the SMV tumor thrombus (maximum standardized uptake value = 45.9). Based on these clinical and imaging findings, malignant lymphoma was considered the leading diagnosis. A CT-guided core needle biopsy was performed, and DLBCL was confirmed histopathologically. Although rare, malignant lymphoma can present with tumor thrombus. Failure to consider malignant lymphoma before biopsy may result in inadequate tissue sampling and inappropriate specimen handling, particularly because fresh tissue is required for immunophenotypic and genetic analyses. This may lead to delayed or inaccurate diagnosis. Therefore, malignant lymphoma should not be excluded from the differential diagnosis solely because tumor thrombus is present.

## Introduction

Diffuse large B-cell lymphoma (DLBCL) is the most common subtype of non-Hodgkin lymphoma and can involve not only lymph nodes but also extranodal lymphatic and extralymphatic tissues [1-3]. Diagnostic imaging plays an important role throughout the clinical course of malignant lymphoma by raising suspicion based on imaging findings, guiding computed tomography (CT)-guided core needle biopsy, contributing to disease staging, and assessing treatment response [1,4-8]. However, atypical imaging findings may delay the diagnosis of malignant lymphoma and consequently postpone appropriate treatment. One such atypical finding is intravascular tumor thrombus.

Tumor thrombus is defined as tumor extension into a vessel and is well recognized in various solid organ malignancies, including hepatocellular carcinoma, renal cell carcinoma, and neuroendocrine tumors [9]. Because lymphomatous masses typically engulf and narrow blood vessels rather than directly invading them, intravascular tumor thrombus is uncommon. Although rare, there have also been several case reports of tumor thrombus in malignant lymphoma [10]. Recognizing this atypical manifestation prior to core needle biopsy is crucial to ensure adequate tissue sampling and proper specimen handling for immunophenotypic and genetic analyses.

We report a case of DLBCL presenting as a mesenteric mass with tumor thrombus extending into the superior mesenteric vein (SMV), together with a review of the relevant literature.

## Case presentation

A 76-year-old female presented to a referring hospital with a one-week history of abdominal pain and a decreased appetite. Other symptoms, including weight loss, vomiting, and constipation, were not observed. Her medical history included hypertension, hyperlipidemia, and gastroesophageal reflux disease. At the referring hospital, a non-contrast CT scan revealed an abdominal mass, and she was referred to our institution for further evaluation. Vital signs on presentation at our hospital were: body temperature of 36.2 °C, blood pressure of 150/66 mmHg, heart rate of 85 beats per minute, and oxygen saturation of 96% on room air. Laboratory investigations revealed hypoalbuminemia, mildly elevated C-reactive protein, and increased levels of both lactate dehydrogenase (LDH) and soluble interleukin-2 receptor (sIL-2R). Tumor markers, including carcinoembryonic antigen and carbohydrate antigen 19-9, were within the normal ranges (Table 1).　

**Table 1:** Laboratory data at initial evaluation. Note: A mildly elevated level of LDH and a markedly elevated level of sIL-2R reflect tumor activity and burden, serving as key laboratory findings that point toward malignant lymphoma. LDH, lactate dehydrogenase; sIL-2R, soluble interleukin-2 receptor

Parameter	On evaluation	Reference range (adult)
Hemoglobin (g/dL)	12.4	11.6-14.8
Platelet count (×10^3^/μL)	484	158.0-348.0
White cell count (×10^3^/μL)	7.81	3.30-8.60
Albumin (g/dL)	3.6	4.1-5.1
Blood urea nitrogen (mg/dL)	10.0	8.0-20.0
Creatinine (mg/dL)	0.48	0.65-1.07
Total bilirubin (mg/dL)	0.3	0.4-1.5
C-reactive protein (mg/dL)	3.31	0.00-0.14
Aspartate aminotransferase (U/L)	15	13-30
Alanine aminotransferase (U/L)	65	10-42
Lactate dehydrogenase (U/L)	252	124-222
Carcinoembryonic antigen (ng/mL)	3.2	0.0-5.0
Carbohydrate antigen 19-9 (U/L)	7	0-37
Soluble interleukin-2 receptor (U/mL)	2190	157-474

Contrast-enhanced CT demonstrated a well-defined, lobulated soft-tissue mass measuring 9 cm in diameter, primarily located within the mesentery along the midline of the lower abdomen (Figure 1). The mass showed heterogeneous enhancement, and a structure suggestive of conglomerated enlarged lymph nodes was identified on its cranial side Furthermore, a tumor thrombus was observed extending from the mass into the SMV and its branching vessels, without involvement of the main portal vein or splenic vein. Notably, there were no secondary signs of bowel ischemia, mesenteric venous congestion, or portal hypertension. Fluorine-18 fluorodeoxyglucose (FDG) positron emission tomography/CT (18F-FDG PET/CT) revealed intense FDG uptake in both the mesenteric tumor and the SMV tumor thrombus (maximum standardized uptake value (SUVmax) = 45.9) (Figure 2). These findings, particularly the bulky mesenteric mass with intense FDG uptake, conglomerate lymphadenopathy, and the elevated LDH and sIL-2R level, raised suspicion for malignant lymphoma. However, the presence of tumor thrombus extending into the SMV was considered atypical for malignant lymphoma and therefore precluded a confident diagnosis. Other differential diagnoses included small intestinal solid tumors, such as neuroendocrine tumors, as well as sarcoma and metastatic tumors.

**Figure 1:**
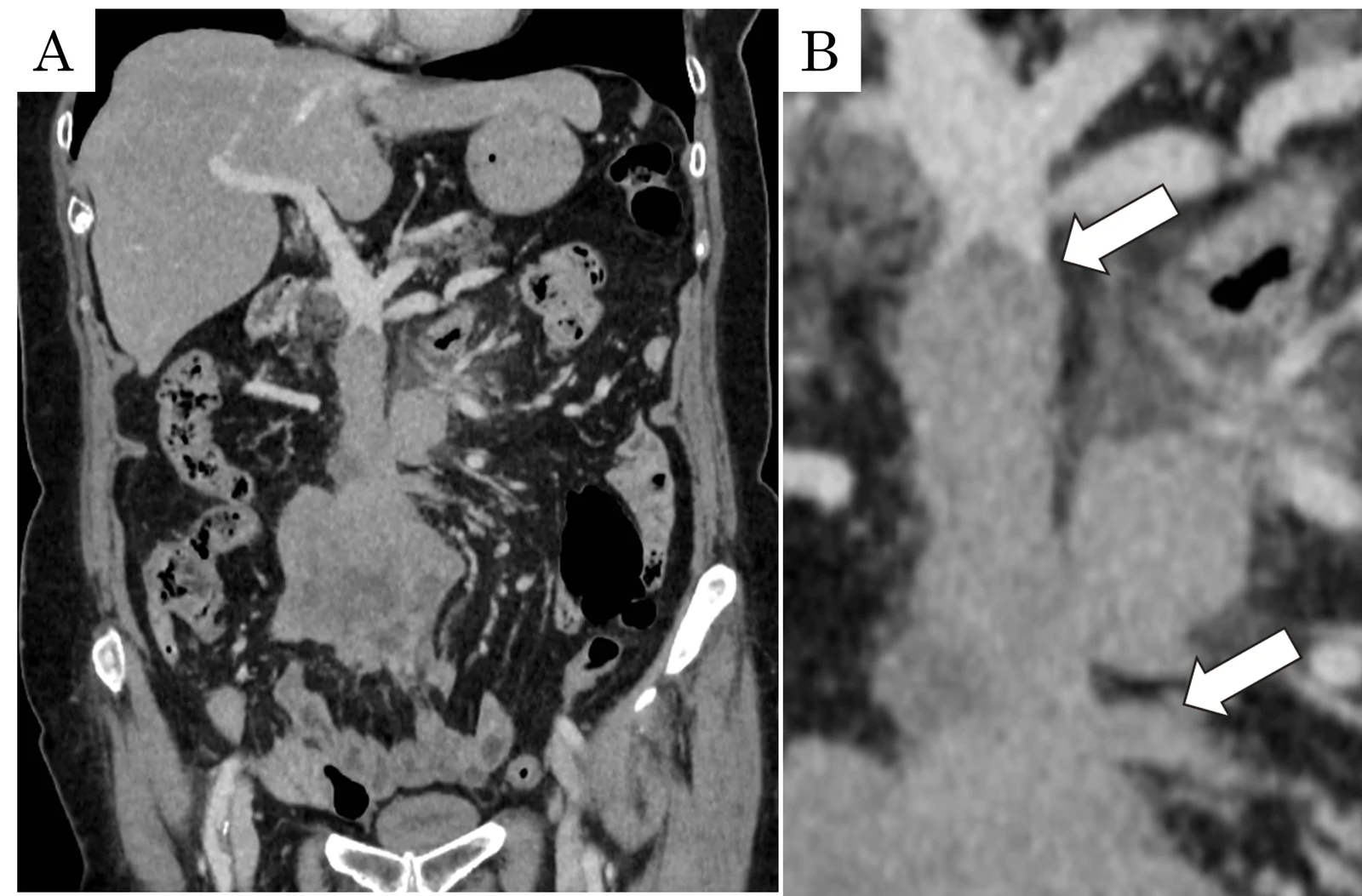
Abdominal contrast-enhanced computed tomography. (A) Coronal portal venous phase image showing a 9-cm lobulated mesenteric mass with heterogeneous enhancement. Conglomerated enlarged lymph nodes are present on the cephalad side of the mass. (B) Enlarged view of the tumor thrombus. An enhancing soft-tissue lesion with attenuation similar to that of the primary tumor extends along the trunk and branches of the superior mesenteric vein (arrows).

**Figure 2:**
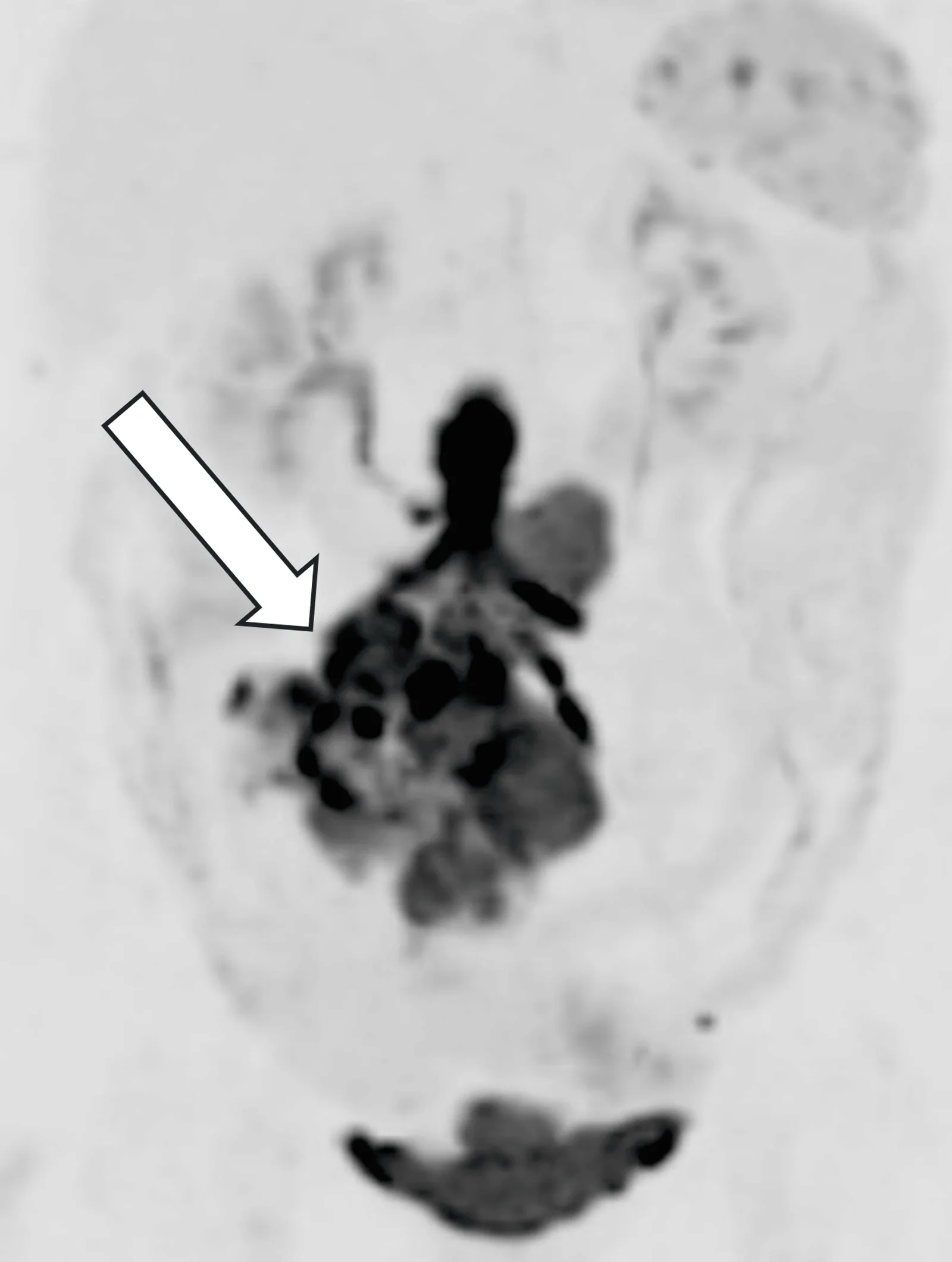
18F-fluorodeoxyglucose positron emission tomography/computed tomography. Maximum intensity projection image demonstrating intense fluorodeoxyglucose uptake in the mesenteric tumor (arrow) and the tumor thrombus within the superior mesenteric vein (maximum standardized uptake value = 45.9).

A CT-guided core needle biopsy was performed three weeks after presentation using an 18-gauge needle for diagnostic purposes. The lower abdominal mesenteric mass was accessed via a route that avoided the bowel and mesenteric arteries and veins, and six tissue specimens were obtained (Figure 3). Four specimens were submitted for histopathological examination, and two were dedicated to flow cytometry and G-band karyotyping. No procedure-related complications, such as active bleeding, occurred.

**Figure 3:**
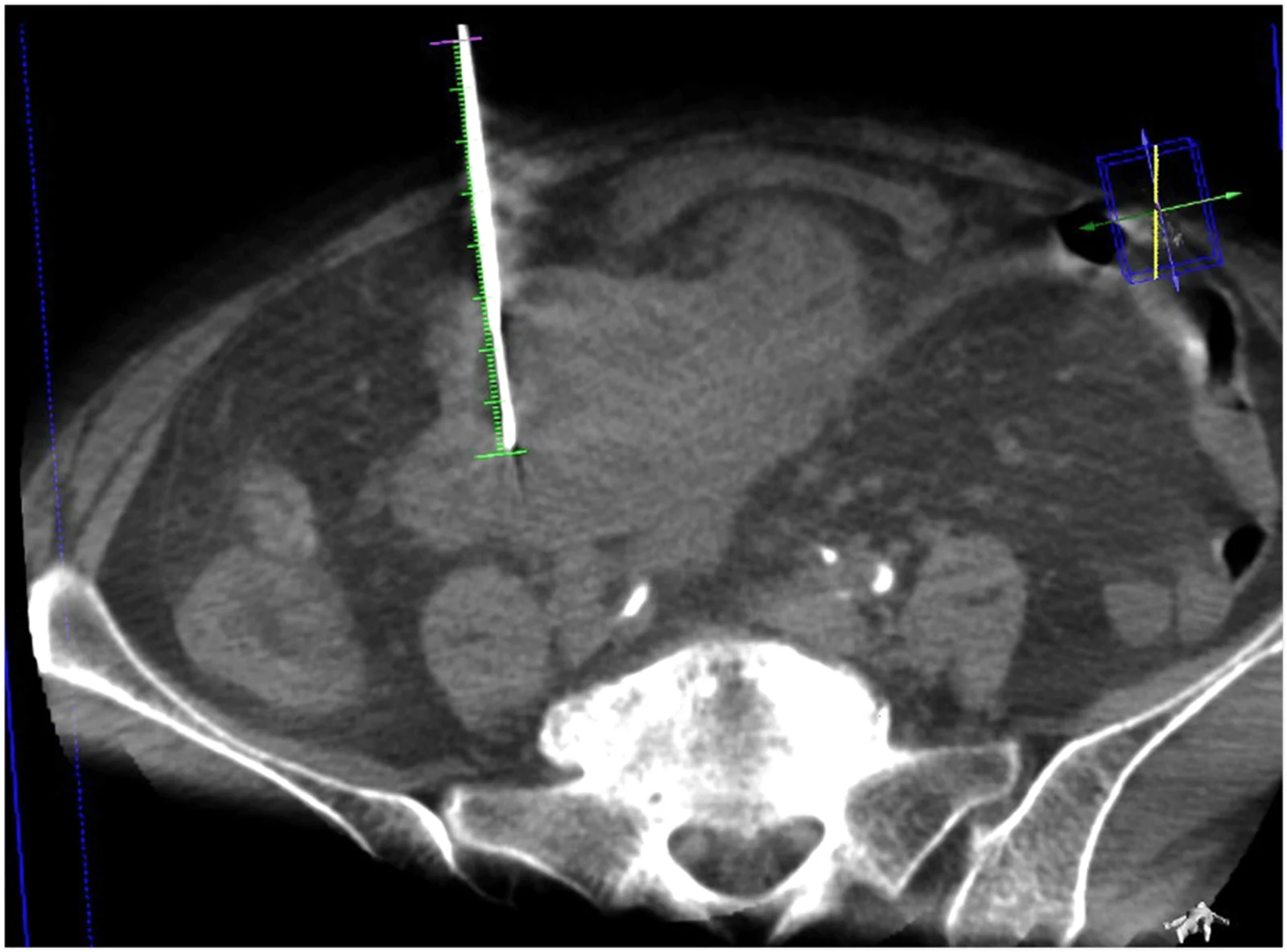
Cone-beam computed tomography image obtained during biopsy. Cone-beam computed tomography-guided core needle biopsy of the lower abdominal mesenteric mass was performed using a trajectory that avoided the bowel and mesenteric arteries and veins.

Histopathological examination revealed tumor cells with a high nucleus-to-cytoplasm ratio proliferating in foci within a vascular stroma, with cord-like infiltration into the fibrous stroma and areas of tumor necrosis (Figure 4). Immunohistochemical staining demonstrated diffuse positivity for CD20, CD79a, CD10, and Bcl-2, with negativity for CD5, whereas CD3 was positive only in the intervening lymphocytes. Flow cytometry revealed a monoclonal λ-restricted B-cell population positive for CD10, while G-band karyotyping failed due to poor cell growth. Based on these findings, a diagnosis of germinal center B-cell-like DLBCL was established one month after the initial presentation.

**Figure 4:**
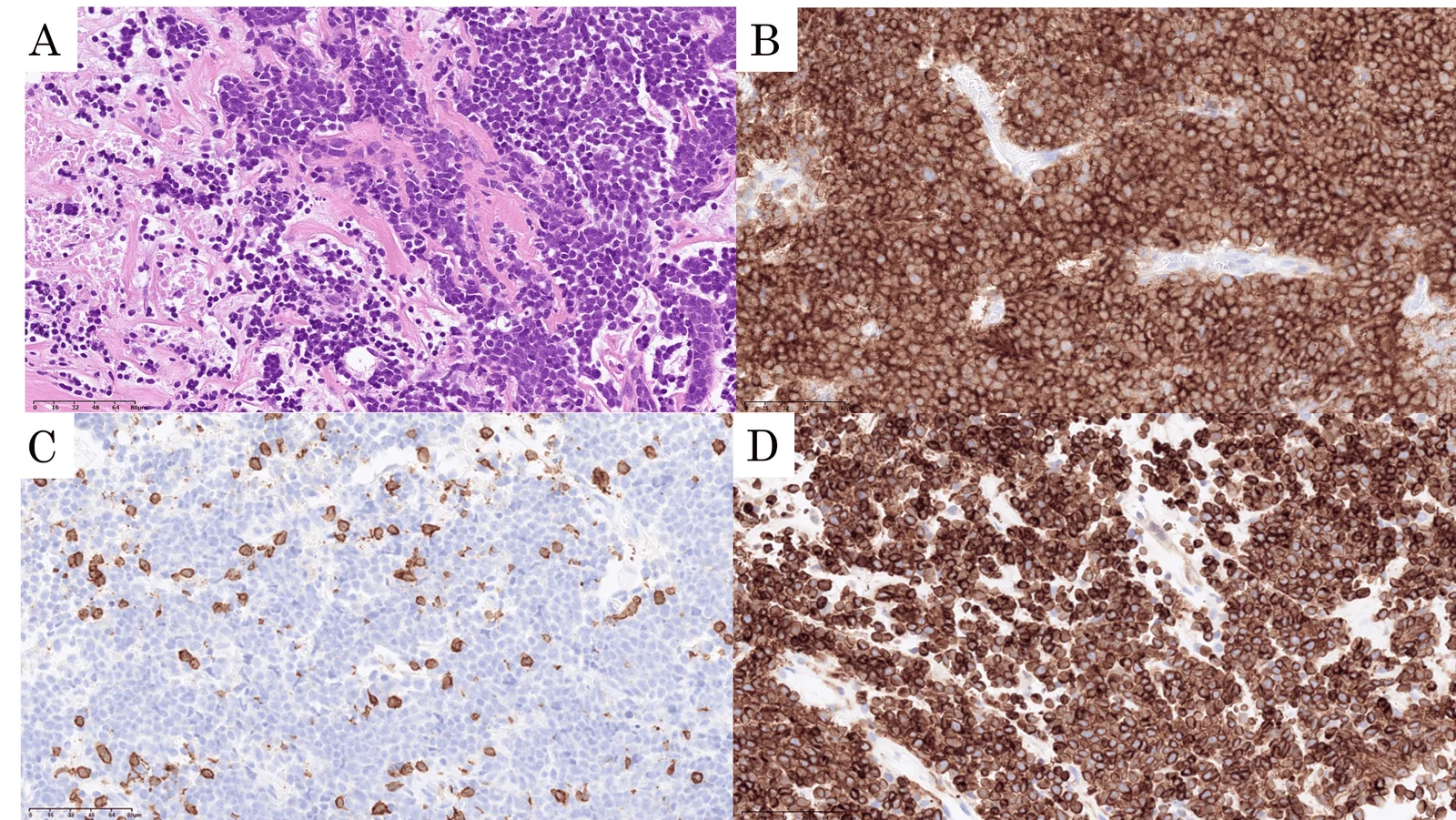
Histopathological findings of the biopsy specimen. (A) Tumor cells with a high nucleus-to-cytoplasm ratio proliferate in foci within a vascular stroma, showing cord-like infiltration into the fibrous stroma and areas of tumor necrosis (hematoxylin and eosin stain; scale bar = 80 µm). (B) Tumor cells showing diffuse positivity for CD20 (scale bar = 80 µm). (C) CD3 positivity is observed only in the intervening lymphocytes (scale bar = 80 µm). (D) Tumor cells showing diffuse positivity for Bcl-2 (scale bar = 80 µm).

The patient was treated with polatuzumab vedotin, rituximab, cyclophosphamide, doxorubicin, and prednisone (Pola-R-CHP). At approximately six months after treatment initiation, the tumor had decreased in size; however, FDG uptake persisted in the SMV lesion, indicating a partial metabolic response [8]. Second-line rituximab, gemcitabine, dexamethasone, and cisplatin (R-GDP) therapy was subsequently initiated, resulting in stable disease as the best response.

## Discussion

This case demonstrates DLBCL presenting with a tumor thrombus extending into the SMV, representing an uncommon and potentially misleading manifestation of malignant lymphoma. Recognition of this atypical presentation is clinically important because it may substantially influence both the diagnostic approach and subsequent management.

Tumor thrombus is classically associated with solid malignancies, including hepatocellular carcinoma, renal cell carcinoma, and neuroendocrine tumors, in which direct intravascular extension is a well-established pattern of disease spread [11-13]. Consequently, the presence of tumor thrombus often leads clinicians to prioritize these entities in the differential diagnosis. In contrast, tumor thrombus in malignant lymphoma is rare and is, therefore, less likely to be considered initially.

In this case, several imaging findings favored malignant lymphoma. The tumor thrombus was contiguous with the mesenteric mass and demonstrated intense FDG uptake comparable to that of the primary lesion, suggesting tumor involvement rather than bland thrombus. In addition, the presence of conglomerated lymphadenopathy further supported the diagnosis of malignant lymphoma. Nevertheless, because tumor thrombus is generally considered an atypical feature of lymphoma, alternative diagnoses, including sarcoma, neuroendocrine tumors, and metastatic disease, could not be excluded.

Malignant lymphoma with tumor thrombus is uncommon, but there have been reports. Chauhan et al. [10] analyzed 14 cases of lymphoma with tumor thrombus, including seven cases of DLBCL, with the portal vein and inferior vena cava being the most frequently involved vessels. Only one case involved the SMV, highlighting the rarity of SMV tumor thrombus in malignant lymphoma. In addition, some reported cases primarily involved extranodal sites, such as the liver or kidneys, necessitating differentiation from solid tumors [14-18].

This case highlights the importance of including malignant lymphoma in the differential diagnosis, even in the presence of tumor thrombus. Failure to consider lymphoma before core needle biopsy may result in a suboptimal biopsy strategy. Unlike most solid tumors, the diagnosis of malignant lymphoma requires not only histopathological evaluation but also immunophenotypic and genetic analyses [1,19,20]. Therefore, when lymphoma is included in the differential diagnosis, it is important to obtain an adequate amount of tissue and allocate specimens appropriately for ancillary studies. Although excisional biopsy is generally recommended for the diagnosis of lymphoma, core needle biopsy remains an important diagnostic tool because of its minimally invasive nature. However, larger or multiple core samples may be required to preserve tissue architecture and obtain sufficient fresh, unfixed material for flow cytometry and molecular analyses [21]. If lymphoma is not suspected before biopsy, inadequate tissue sampling and inappropriate specimen processing may compromise diagnostic accuracy and delay definitive diagnosis.

From a practical perspective, radiologists and clinicians should carefully evaluate imaging findings suggestive of lymphoma, such as homogeneous or mildly heterogeneous soft-tissue masses, associated lymphadenopathy, and extranodal involvement, even in the presence of tumor thrombus. When lymphoma is considered a diagnostic possibility, biopsy planning should be optimized to ensure adequate tissue acquisition and appropriate specimen allocation. Overall, this case underscores that the presence of tumor thrombus does not exclude malignant lymphoma and that awareness of this rare presentation is essential for accurate diagnosis and appropriate management.

## Conclusions

We report a case of DLBCL with a tumor thrombus involving the SMV. Although rare, malignant lymphoma can present with tumor thrombus, and this possibility should be recognized in clinical practice. Failure to consider malignant lymphoma before biopsy may result in inadequate biopsy tissue and inappropriate specimen handling, potentially leading to insufficient diagnostic information and delays in diagnosis and treatment. As this case illustrates, malignant lymphoma should not be excluded from the differential diagnosis solely because a tumor thrombus is present.

## References

[1] Sehn LH, Salles G (2021). Diffuse large B-cell lymphoma. N Engl J Med.

[2] Alaggio R, Amador C, Anagnostopoulos I (2022). The 5th edition of the World Health Organization classification of haematolymphoid tumours: lymphoid neoplasms. Leukemia.

[3] Kurz KS, Ott M, Kalmbach S (2023). Large B-cell lymphomas in the 5th edition of the WHO-classification of haematolymphoid neoplasms-updated classification and new concepts. Cancers (Basel).

[4] Barrington SF, Mikhaeel NG, Kostakoglu L (2014). Role of imaging in the staging and response assessment of lymphoma: consensus of the International Conference on Malignant Lymphomas Imaging Working Group. J Clin Oncol.

[5] Casasnovas R-O, Meignan M, Berriolo-Riedinger A (2011). SUVmax reduction improves early prognosis value of interim positron emission tomography scans in diffuse large B-cell lymphoma. Blood.

[6] Frampas E (2013). Lymphomas: Basic points that radiologists should know. Diagn Interv Imaging.

[7] Hesselmann V, Zähringer M, Krug B, Wesselmann C, Haferkamp K, Wickenhauser C, Lackner K (2004). Computed-tomography-guided percutaneous core needle biopsies of suspected malignant lymphomas: impact of biopsy, lesion, and patient parameters on diagnostic yield. Acta Radiol.

[8] Cheson BD, Fisher RI, Barrington SF (2014). Recommendations for initial evaluation, staging, and response assessment of Hodgkin and non-Hodgkin lymphoma: the Lugano classification. J Clin Oncol.

[9] Quencer KB, Friedman T, Sheth R, Oklu R (2017). Tumor thrombus: incidence, imaging, prognosis and treatment. Cardiovasc Diagn Ther.

[10] Chauhan A, Garg N, Menias CO, Devine CE, Bhosale PR, Balachandran A (2015). Tumor thrombus as a rare presentation of lymphoma: a case series of 14 patients. AJR Am J Roentgenol.

[11] Elbanna KY, Khalili K, O'Malley M, Chawla T (2020). Imaging and implications of tumor thrombus in abdominal malignancies: reviewing the basics. Abdom Radiol (NY).

[12] Tathireddy H, Rice D, Martens K, Shivakumar S, Shatzel J (2023). Breaking down tumor thrombus: current strategies for medical management. Thromb Res.

[13] Yedururi S, Kang H, Cox VL, Chawla S, Le O, Loyer EM, Marcal L (2019). Tumor thrombus in the venous drainage pathways of primary, recurrent and metastatic disease on routine oncologic imaging studies: beyond hepatocellular and renal cell carcinomas. Br J Radiol.

[14] Lim HJ, Park M-S, Kim Y-E (2020). Diffuse large B-cell lymphoma in the portal vein. J Korean Soc Radiol.

[15] Chen L, Richendollar B, Bunting S, Campbell S, Zhou M (2013). Lymphomas and lymphoproliferative disorders clinically presenting as renal carcinoma: a clinicopathological study of 14 cases. Pathology.

[16] Matsumoto S, Mori H, Takaki H, Ishitobi F, Shuto R, Yokoyama S (2004). Malignant lymphoma with tumor thrombus in the portal venous system. Abdom Imaging.

[17] Natsuizaka M, Kudo M, Suzuki M (2009). Diffuse large B-cell lymphoma with massive portal vein tumor thrombosis in a patient with alcoholic cirrhosis: a case report and literature review. Intern Med.

[18] Kanemura N, Tsurumi H, Yamada T, Kojima Y, Kasahara S, Moriwaki H (2006). Diffuse large B-cell lymphoma with tumor thrombosis in the portal vein. Int J Hematol.

[19] Kronland R, Grogan T, Spier C (1985). Immunotopographic assessment of lymphoid and plasma cell malignancies in the bone marrow. Hum Pathol.

[20] Li S, Jaye DL, Bradley KT (2017). Multimodality technologies in the assessment of hematolymphoid neoplasms. Arch Pathol Lab Med.

[21] Syrykh C, Chaouat C, Poullot E (2022). Lymph node excisions provide more precise lymphoma diagnoses than core biopsies: a French Lymphopath network survey. Blood.

